# DNA metabarcoding suggests dietary niche partitioning in the Adriatic European hake

**DOI:** 10.1038/s41598-022-05346-0

**Published:** 2022-01-25

**Authors:** Giulia Riccioni, Marco Stagioni, Chiara Manfredi, Fausto Tinti, Corrado Piccinetti, Simone Libralato

**Affiliations:** 1 Department of Biological, Geological and Environmental Sciences, Laboratory of Marine Biology and Fisheries, University of Bologna, viale Adriatico 1/n, 61032 Fano, Italy; 2 Co.N.I.S.Ma- URL Fano, Laboratory of Marine Biology and Fisheries, viale Adriatico 1/n, 61032 Fano, Italy; 3 Department of Biological, Geological and Environmental Sciences, University of Bologna, via Sant’Alberto 163, 48123 Ravenna, Italy; 4 National Institute of Oceanography and Applied Geophysics - OGS, Via Beirut 2/4 (Ex-Sissa building), 34151 Trieste, Italy; 5 BioDNA - Centro di Ricerca sulla Biodiversità e DNA Antico, Facoltà di scienze Agrarie, Alimentari e Ambientali, Università Cattolica del S. Cuore, via Emilia Parmense n. 84, 29122 Piacenza, Italy

**Keywords:** Ecology, Evolution

## Abstract

The Northern Adriatic Sea (FAO Geographical Sub-Area 17) is one of the most productive fishing areas of the Mediterranean Sea and it includes a broad diversity of habitats. In the Northern Adriatic basin, the Pomo Pit (200–273 m of depth) is one of the most important areas of aggregation for some demersal stocks shared in the Adriatic Sea and it is an important spawning/nursery area of the European hake (*Merluccius merluccius*). Through a metabarcoding approach we investigated the feeding habits of European hake, both inside and outside the Pomo Pit, and their temporal variability comparing samples collected in 2016 and 2014. Our analyses proved the presence of an ontogenetic shift from a diet based mainly on crustaceans in juveniles to a more piscivorous feeding behaviour in adult hakes and suggested the presence of a specific niche partitioning and food preferences between hakes living inside and outside the Pomo Pit. The main differences among adult hakes refer to the presence of molluscs in the stomachs of hakes collected within the Pomo Pit and the presence of high depth prey species (i.e., *Micromesistius poutassou*). Metabarcoding revealed the relevant ecological role played by the Pomo Pit in *M. merluccius* feeding behaviour and ontogenetic development, promoting a careful ecosystem-based management of fisheries in this area through focused conservation measures.

## Introduction

Spatial heterogeneity in geomorphological and oceanographic variables plays a key role in marine ecosystems by influencing population structure, species composition and several ecological processes^1–3^. Local environmental features, particular types of habitats together with fishing effort can drive the partitioning of spatio-temporal aggregations of demersal and benthopelagic species within complex faunal assemblages^4^. Fishing pressure on apex predators can deeply alter such partitioning and food webs and heavy top-down effects should be carefully considered to implement fishery management plans^5–8^.

The Northern Adriatic Sea (i.e., the FAO Geographical Sub-Area 17) is one of the most productive areas of the Mediterranean Sea including a broad diversity of habitats, such as rocky and soft bottoms, large estuaries and lagoons, seagrass meadows and deep water environments^9–11^. It is one of the major fishing grounds in the Mediterranean Sea^12^. Adriatic fisheries play an important socio-economic role in the coastal communities for all countries surrounding the basin^13^. In the last decades bottom trawling in the Adriatic Sea has been particularly intense leading to the depletion of key demersal species. Within the basin, the Pomo Pit (Jabuka in Croatian, 200–273 m of depth) is one of the most important habitats for several demersal stocks^14–16^. The peculiar physical and oceanographic characteristics support the presence of Essential Fish Habitats (EFH) in this area^12^ for high-priority fish and invertebrate species^17^. The Northern Adriatic is considered a sensitive and critical zone^18–20^ for the presence of an important spawning/nursey area for the stock of European hake. Indeed, a spatial distribution model described the Pomo area as a hot spot of biomass density in the Mediterranean^21^. In 2016 the General Fisheries Commission for the Mediterranean discussed the adoption of memorandum of understanding for Vulnerable Marine Ecosystems and extensive national efforts to monitor and ban fishing from the area were made^22^. In 2017 the GFCM established the temporary Fishery Restricted Area in the Pomo Pit regulating demersal fishery and other fishing activities in the Pomo area (REC.CM GFCM/41/2017/3). Currently, pressure is exerted on the European Commission by more than 20 Non-Governmental Organizations to permanently protect the Pomo Pit^23^.

The European hake is a nektobenthic apex predator of shelf and upper slope communities showing a very wide depth range (20–1000 m). It spreads over the whole Mediterranean Sea and in the southern part of the North-eastern Atlantic^24^. The European hake is one of the most high-priority target species for the Mediterranean trawl fisheries and recent assessments indicated the Mediterranean and Black Sea stocks heavily overfished^12^. Current levels of fishing mortality of the Adriatic stock are estimated as 2.78 times higher than FMSY (Fishing pressure that gives the Maximum Sustainable Yield in the long term)^12^. From an ecological point of view, *M. merluccius* is one of the most abundant and fast-growing fish predators of the Mediterranean and its predatory feeding behaviour strongly impacts other species in the ecosystem. European hake can have, indeed, a relevant role in terms of biomass removal of their prey^25–27^; for this reason a good understanding of its feeding habits is crucial to know implication of fishing management measures. In the Northern Adriatic Sea, European hake juveniles (Total Length, TL < 120 mm) are mainly restricted to the Pomo Pit area as a consequence of their limited mobility and usually their diet is quite different from adults because of their smaller size and the different environment inhabited^28^. Along its distribution range several studies have been performed to provide information about its dietary habits^25,27,29,30^ (Supplementary Table S1). DNA metabarcoding has been extensively applied and has proven to be an effective tool for characterizing the diet of marine macrofauna^31–34^. Recently, we set up and tested a molecular genetic method to provide an in-depth description of *M. merluccius* food preferences in the Adriatic Sea improving the taxonomic identification of prey compared to the classical morphological identification in stomach content^35^. This metabarcoding method allowed us the identification of a broad range of prey for *M. merluccius* highlighting different food preferences by size classes. Nonetheless specimens of the smallest size class (TL < 120 mm) were not included in the previous analysis^35^ preventing the detection of the characteristic ontogenetic shift in hake diet^36,37^. However, the analysis of critical and heterogeneous habitats, as the nursery areas, is decisive when dealing with apex predators which can have a strong effect in shaping spatial biodiversity patterns^8^. Considering the pressure currently exerted to permanently close Pomo Pit area, the knowledge on the ontogenetic diet shifts associated with the nursery area could further stress the ecological importance of the Pomo Pit and could support the temporal extension of the spatial management measures temporarily in place.

This study tests whether ecosystem and environmental conditions partitioned the feeding habits and affected food webs, ontogeny and prey selectivity of *M. merluccius* in the Northern Adriatic Sea, by comparing the feeding behaviour of hake specimens caught inside and outside the hake nursery area located in the Pomo Pit before the establishment of the Fishery Restricted Area (FRA). We assessed prey species composition by metabarcoding analysis of stomach content of 100 hake specimens collected both in 2014 and in 2016 and categorized according to size classes and habitats. We tested the impact of environmental and ecological drivers on the diet preferences of European hake in the area by wide and robust statistical analyses. Results corroborate the critical ecological role that the Pomo Pit plays for the European hake in the Adriatic Sea, supporting the maintenance of fisheries restrictions in order to permit sustainable exploitation of this and other exploited marine resources.

## Results

**Figure 1:**
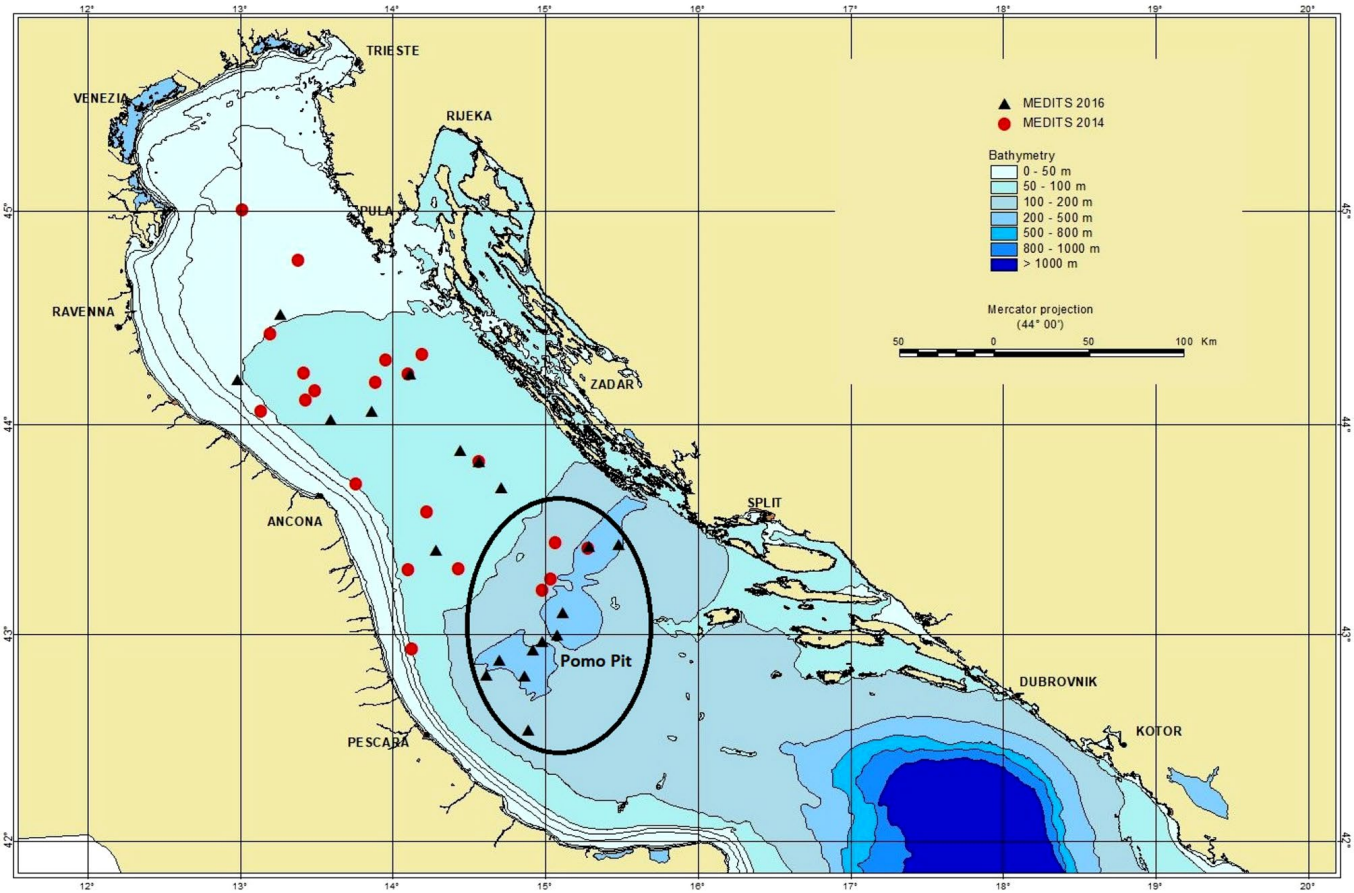
Map of the sampling hauls in the NC Adriatic Sea: triangles depict samples collected in 2016, circles samples collected in 2014. Further details can be found in Supplementary Material Table S1. This map was created using ArcViewGIS version 3.2a (https://geonet.esri.com/thread/36365). The Adriatic cartography used is freely available at http://www.faoadriamed.org/html/adr_inf_centre.html#cart.

MiSeq sequencing produced about 18 million of reads from the stomach content DNA of 100 *M. merluccius* specimens collected both inside the Pomo Pit and outside on the shelf area (Fig. 1, see “Methods” for sampling description). After bioinformatic analyses 1,371 OTUs were obtained. The similarity search analysis of stomach content DNA sequences detected 32 prey species both shared or exclusive of the two different habitats (inside and outside the Pomo Pit, Table 1). The adequacy of stomach sample sizes was assessed by generating accumulation curves (with rarefaction method) of species recorded per stomach sample (Supplementary Fig. S1). The two accumulation curves obtained considering the two sampling areas are approaching the plateau (Supplementary Fig. S1) and the species richness values were similar for both the sites and were comparable to the values obtained in Riccioni et al.^29^, which included only specimens sampled outside the Pomo Pit.

**Table 1:** List of the species and families identified in stomach content samples collected within and outside the Pomo Pit.

Species	Family	Within	Outside
*Alpheus glaber*	Alpheidae	**×**	**×**
*Chlorotocus crassicornis*	Pandalidae	**×**	**×**
*Euphausia krohni*	Euphausiidae	**×**	
*Halice walkeri*	Pardaliscidae		**×**
*Meganyctiphanes norvegica*	Euphausiidae	**×**	
*Nematoscelis megalops*	Euphausiidae	**×**	
*Parapenaeus longirostris*	Penaeidae	**×**	
*Philocheras bispinosus*	Crangonidae		**×**
*Processa nouveli*	Processidae		**×**
*Solenocera membranacea*	Solenoceridae		**×**
*Eledone moschata*	Octopodidae	**×**	**×**
*Illex coindetii*	Ommastrephidae	**×**	
*Rondeletiola minor*	Sepiolidae	**×**	
*Todarodes sagittatus*	Ommastrephidae	**×**	
*Todaropsis eblanae*	Ommastrephidae	**×**	
*Arnoglossus laterna*	Bothidae		**×**
*Chelidonichthys cuculus*	Triglidae	**×**	
*Engraulis encrasicolus*	Engraulidae	**×**	**×**
*Gadiculus argenteus*	Gadidae	**×**	
*Gaidropsarus macrophthalmus*	Gadidae		**×**
*Lesueurigobius friesii*	Gobiidae		**×**
*Maurolicus muelleri*	Sternoptychidae	**×**	
*Merlangius merlangus*	Gadidae	**×**	
*Micromesistius poutassou*	Gadidae	**×**	
*Mullus barbatus*	Mullidae		**×**
*Sardina pilchardus*	Clupeidae		**×**
*Scorpaena notata*	Scorpaenidae		**×**
*Serranus hepatus*	Serranidae		**×**
*Trachurus trachurus*	Carangidae	**×**	**×**
*Trisopterus capelanus*	Gadidae	**×**	
*Raja miraletus*	Rajidae		**×**
*Gracilechinus acutus*	Echinidae		**×**

**Figure 2:**
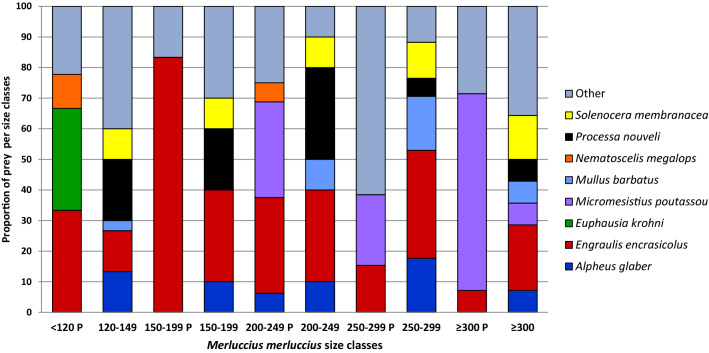
Comparison of dietary richness among *M. merluccius* size classes inside and outside the Pomo Pit: frequency occurrence data of species are reported (based on the number of reads). The eight most recurrent items plus “other” items across all classes are shown. Samples depicted with a P have been sampled inside the Pomo Pit.

The ranking by occurrence of prey species in the two habitats showed that *Engraulis encrasicolus* was present in all the stomachs (Fig. 2, Supplementary Table S2). In the Pomo Pit, large-sized hake individuals showed a size-related preference for *Micromesistius poutassou*, (Fig. 2, size classes TL 200–249 P, TL 250–299 P and ≥ 300 P) while small-sized hakes (TL < 120 P) preferred *Euphausia krohni* (known to be the most abundant species of krill in the area). Notably three species of decapods (*Processa nouveli*, *Solenocera membranacea* and *Alpheus glaber*) and a teleost (*Mullus barbatus*) were recurrent in all the five classes collected outside the Pomo Pit (Fig. 2). These recurrent species were also identified in the previous survey^35^ suggesting that, at least for the shallower habitat, *M*. *merluccius* diet is stable in time. *Lesuerigobius friesii*, which showed a high occurrence in stomachs sampled in 2014 (size classes TL 120–149 and TL 150–199) and was detected as an indicator species for the smaller size class^35^, in this survey showed to be rare in the diet.

Overall, Bray–Curtis dietary pairwise inter-class size dissimilarities (Table 2) ranged from 0.263 (TL 150–199 S *vs.* TL 200–249 S) to 0.955 (TL 120–149 P *vs.* TL ≥ 300 P). Within the Pomo Pit, the smallest (TL < 120 P and TL 150–199 P) and largest (TL ≥ 300 P) size classes showed Bray–Curtis dissimilarity values at ~ 0.9, i.e., the highest among those computed between size-classes within the area (upper left part of Table 2) and higher than those computed within the size class (Supplementary Table S3). Outside the Pomo Pit, the inter-size class values (bottom-right part of Table 2) are quite low (none is higher than 0.7) and are all lower than corresponding within-size class values (see Table S3). The inter-class size dissimilarity values observed between samples collected within and outside the Pomo Pit ranged from 0.565 to 0.955 (bottom-left part of the Table 2), with a mean value of 0.759. The largest size classes sampled within the Pomo Pit (TL 250–299 P and TL ≥ 300 P) showed high dissimilarities with all size classes outside Pomo Pit (dissimilarity values > 0.8; Table 2). The mean values of the inter-size class dissimilarity computed within each habitat (within and outside the Pomo Pit) were P: 0.677 and S: 0.45, while the corresponding mean within-size class values were P: 0.53 and S: 0.73 (Supplementary Table S3).

**Table 2:**
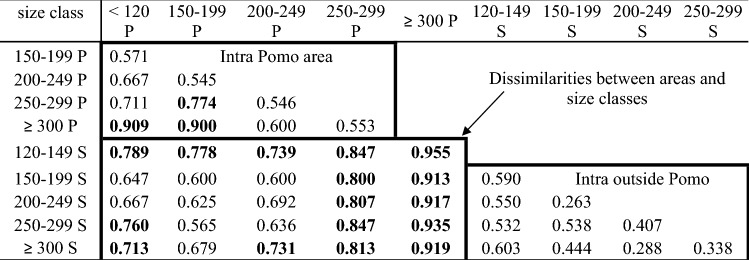
Inter-size class variability measured as Bray–Curtis dissimilarities calculated using occurrence-based data for individuals sampled in Pomo Pit area (P) and outside (S).

Values > 0.7 are bold faced. Frames highlight intra area dissimilarities (both for Pomo Pit and outside) and between areas dissimilarities.

**Figure 3:**
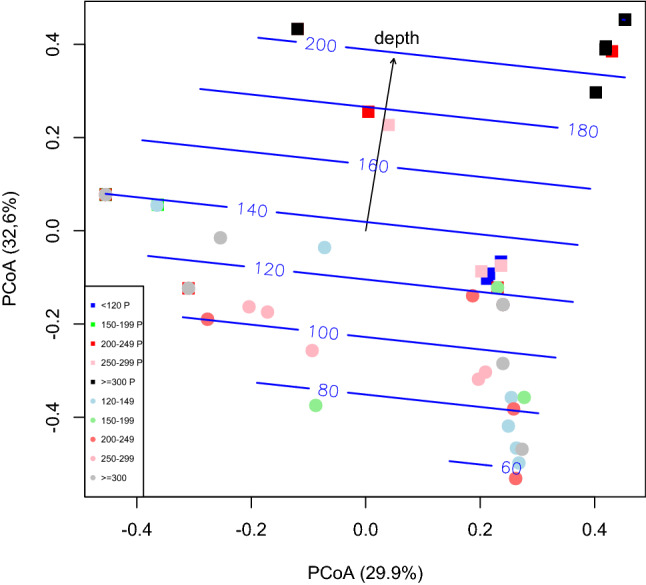
Principal Coordinate Analysis based on Bray–Curtis dissimilarities computed on the occurrence data standardised by Hellinger transformation of samples from all size classes and habitats (permutest *P*-value = 0.004). Samples collected inside the Pomo Pit (identified in the legend with a P) are depicted as squares, outside the Pomo Pit as dots. The vector and the linear trend surface computed by using sea depth as environmental variable are shown on the PCoA plot (numbers on the splines represent depth values measured in meters). Plot made by using the R program^77^.

The Principal Coordinate Analysis (PCoA) plot (Fig. 3), based on the Bray–Curtis dissimilarities computed on Hellinger transformed occurrence data, showed a significant clustering among the size classes (permutest *P*-value = 0.004). An important dietary differentiation of the size class TL 150–199 was apparent, resulting significantly differentiated from most of the other samples collected in the Pomo Pit and from all the size classes collected outside the Pomo Pit (revealed by the significant comparisons *P*-value ranging between 0.001 and 0.009, Supplementary Table S4). A certain level of dietary partitioning was detected for the largest size class from the Pomo Pit (TL ≥ 300 P), which gave 1%- and 5%-significant pairwise comparisons with most of the other size classes. Interestingly the PCoA plot clearly separated the two small-sized classes within the Pomo Pit from the large-sized classes that clustered together on the top of the plot (pink, red and black squares, Fig. 3). On the contrary, all the samples collected outside the Pomo Pit, at shallower depth, clustered together showing a slight differentiation among size classes (light-blue and grey dots, Fig. 3). This result suggested the presence of a relationship between the pattern of differentiation among the size classes and depth also supported by the ANOSIM analysis (r^2^ = 0.27, *P*-value  <  < 0.01) and the permutational PERMANOVA analysis (r^2^ = 0.33, *P*-value <  < 0.01) showing that the tested grouping accounted for about a 30% of the variance present in the data.

The PERMANOVA analysis also identified the habitat as a factor significantly driving feeding behaviour (r^2^ = 0.11, *P*-value <  < 0.01) and when considering the interaction between size classes and habitat (r^2^ = 0.33, *P*-value <  < 0.01). Moreover, the vector computed by using depth as environmental variable (*envfit* analysis) showed a significant *P*-value (*P*-value = 0.001) and a r^2^ = 0.47 (Fig. 3).

**Figure 4:**
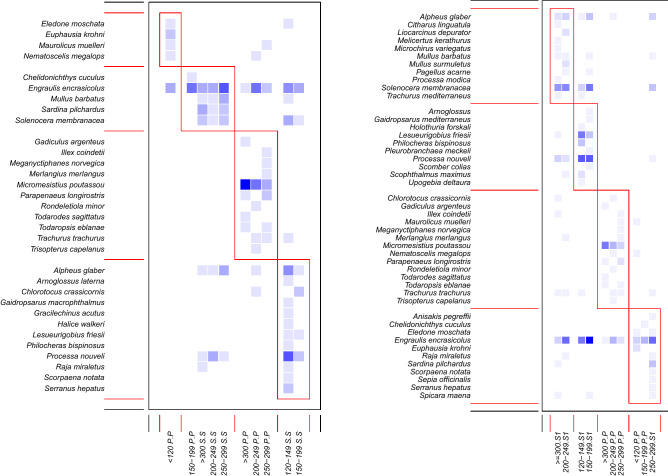
Heat map showing modules (aggregated sets of interacting species) detected in the bipartite weighted graphs by applying Newman’s modularity measure. Shading of matrix entries indicates number of observations (darker shades describe a higher number of observations). On the left modules identified in 2016 samples (inside Pomo, P; shelf outside S), on the right modules identified by using samples collected within the Pomo Pit in 2016 (P) and outside the Pomo Pit in 2014 (depicted as S1, Riccioni et al*.*, 2018). Modules are identified in red. Analysis performed by using the R package *bipartite*^75^ and R environment^77^.

The bipartite network depicting diet composition (Supplementary Fig. S2, on the top) showed a higher complexity of prey-predator relationships among the *M*. *merluccius* samples collected at shallower depth. In the samples collected within the Pomo Pit the number of links per size class was 1.25 whereas in the samples collected at shallower depth the number of links per size class was 1.78 Moreover, the weighted connectance was 0.16 in the deep samples and 0.25 in the shallow samples suggesting the presence of a constraint in the diet for *M. merluccius* individuals sampled inside the Pomo Pit and of a narrower spectrum of preys in this deep area. The weighted NODF value was lower in the Pomo Pit sample (17) than in the shallower sample (31) revealing that some size classes are characterized by a higher degree of niche overlap outside the Pomo Pit confirming previous results^35^. Distribution density of prey among the *M. merluccius* size classes was displayed by using a heat map of the network (Supplementary Fig. S2) which gave a simple visualisation of the species detected. The heat map also showed the presence of a higher prey diversity in the samples collected outside the Pomo Pit confirming the bipartite network result (Supplementary Fig. S2). The module analysis detected four modules (link-rich clusters of species in a community) by clustering the samples collected within the Pomo Pit in two modules: one for the smallest size class and one for the largest size classes together (except 150–199 P, that clustered with samples from the shallow area outside Pomo); a similar clustering separated large (200–249 S; 250–299 S; > 300 S) and small size classes (120–149 S; 150–199 S) for shallower sample (Fig. 4 on the left). Performing the same module analysis by using *M. merluccius* metabarcoding stomach content data collected in 2014 and the samples collected within the Pomo Pit in 2016 (Fig. 4 on the right) provided exactly the same number of modules quite similar: in this case large (200–249 S1; > 300 S1) and small size classes (120–149 S1; 150–199 S1) from 2014 aggregated together; the two smallest size-classes in Pomo (< 120 P; 150–199 P) aggregated together but also with an intermediate size class outside Pomo (250–299 S from 2014). Modules showed similar grouping of large and small size-classes by area with great consistency of size-class and species aggregation among 2016 and 2014 samples, although some reshuffling of species was resulting.

**Figure 5:**
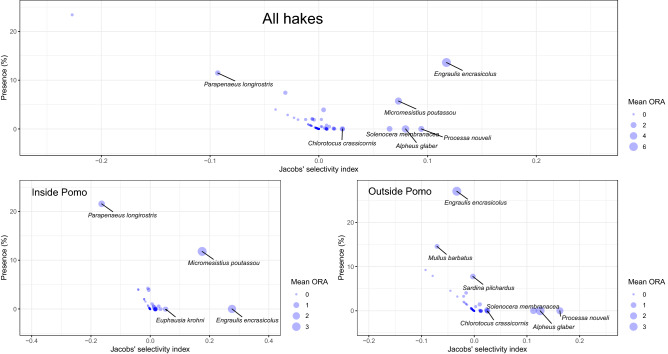
Phylum trophic significance based on presence (%), on individuals/km^2^, on mean ORA (%) in stomach samples and Jacobs’ selectivity index. Species with a mean ORA > 1 are shown. Analysis performed by using the R package *selectapref*^80^ in R environment^77^.

Jacobs’ selectivity index was computed for all the *M. merluccius* size classes together and for each distinct habitat (Fig. 5) considering the mean ORA and prey abundance indices (as individuals/km^2^) obtained from MEDITS trawl survey as a proxy for the relative density at sea (Fig. 5). When pooling all stomach data together, positive values for Jacobs’ index represent preferred species and were recorded for *E. encrasicolus*, *M. poutassou*, *A. glaber*, *P. nouveli* and *Chlorotocus crassicornis* (Fig. 5). A negative value was recorded for *Parapenaeus longirostris* although its percentage of individuals per km^2^, considering all the hauls collected, was relatively high (> 10%). Moreover, a cloud of less abundant species showed values of the index around zero suggesting an opportunistic consumption of these prey. When considering the Jacobs’ selectivity index computed only on the samples collected within the Pomo Pit (Fig. 5) the pattern was very similar with high selectivity for *E. encrasicolus* and low selectivity for *P. longirostris*, respectively, linked to low (almost zero) and high value of individuals per km^2^ (> 20%) found in the MEDITS hauls for this area. The Jacobs’ selectivity index analysis computed on metabarcoding data for stomachs collected outside the Pomo Pit confirmed a clear feeding preference for *S. membranacea*, *A. glaber* and *P. nouveli* in spite of the low abundance detected (Fig. 5).

## Discussion

In this study we provide new insights on the trophic ecology of *M. merluccius* in the Northern Adriatic by applying a metabarcoding approach previously tested^35^ to stomach contents of European hake.

Our metabarcoding analyses provided crucial insights into *M. merluccius* feeding behaviour in very heterogeneous habitats and unprecedented fine scale descriptions of trophic relationships within a nursery area currently managed as Fisheries Restricted Area since 2018.

### Selectivity and opportunistic feeding behaviour of European hake

When considering food item preferences or avoidances, some habitat specific pattern can be easily identified. A clear preference for *E. encrasicolus* and *M. poutassou* was evident in *M. merluccius* collected within the Pomo Pit, whereas a preference for decapods emerged in shallower habitats. A negative value of Jacobs’ selectivity index for *P. longirostris*, advocated for the presence of a prey avoidance behaviour despite its high abundance in the Pomo Pit. A common prey species among all the size classes and habitats was *E*. *encrasicolus*. However, a decreasing trend in the abundance of *E. encrasicolus* in the diet of hake was evident from the smallest to the largest size classes collected within the Pomo Pit, in which this prey seemed to be replaced by *M*. *poutassou*. Due to habitat and depth preferences, *E. encrasicolus* shows a very limited distribution in the Pomo Pit, and its presence in stomachs supports the hypothesis of high selectivity for this prey species by European hake. This is in agreement with previous findings based both on the classical morphological and on the metabarcoding approach^25,30,35,36,38–42^. However, the combined high Jacobs’ selectivity index and high-density indices for both *E*. *encrasicolus* and *M*. *poutassou* (Fig. 5) suggest an opportunistic shift in the diet related to prey abundances.

Several other preys were specific of a sampling area thus allowing for an easy differentiation of samples collected at different bathymetries. In the Pomo Pit, some prey species identified by the metabarcoding analysis in the stomach content of *M. merluccius* are typically distributed at higher depth (e.g. *Euphausia krohni*, *Meganyctiphanes norvegica*, *Todaropsis eblanae* and *Micromesistius poutassou*;^43–46^). The list of prey detected clearly showed an almost exclusive presence of cephalopods (*Todarodes sagittatus*, *T. eblanae*, *Rondeletiola minor*, *Illex coindetii*^47^) in the samples collected within the Pomo Pit, where their abundance at sea is higher than outside the area, confirming a general opportunistic feeding behaviour.

In general the samples collected outside the Pomo Pit displayed a large prevalence of decapods ^35,48^, and a growing abundance of fish in the largest size classes. In gut content of specimens collected at shallower depth, in fact, metabarcoding data showed a diet mainly composed of crustaceans and fish, with a high diversity of prey present at high frequency and three species (e.g., *Alpheus glaber*, *Processa nouveli*, *Solenocera membranacea*;^49–51^) were detected in diet of hake at high frequency also in the previous study^35^. A general lower diversity of species was detected in the stomach content from metabarcoding data of the samples collected in the Pomo Pit, which was characterised by the presence of few species with high frequency. These findings on one side are corroborating the current knowledge about the spatial distribution of these prey species in the Northern Adriatic Sea and on the other gave a first glance on the opportunistic feeding behaviour of the European hake. The opportunistic behaviour is quantitatively stressed by the low values of Jacobs’ selectivity index for several species (Fig. 5) and it is also at the base of the influence of the depth on diet of hake, as highlighted by the PCoA ordination overimposed with the depth vector (Fig. 3).

### Spatial differences in ontogenetic dietary shift

The largest size class (TL ≥ 300 P) showed a diet composed almost only by fish with rare molluscs (Supplementary Table S2) confirming the known ontogenetic shift from a diet based mainly on euphasiids in the younger hakes (TL < 120 P) to a diet based mainly on fish for the largest size classes^36^. The intermediate size classes exhibited a wider range of preys with also the presence of crustaceans, revealing a more diverse feeding behaviour in these size classes.

The comparison of the samples collected inside and outside the Pomo Pit suggested a differentiation of niches related to the presence/abundance of different prey species in the two habitats. The size classes sampled at shallower depth fed mainly on fish and crustaceans, and also the largest size class showed a good presence of crustaceans in the diet. We also detected a partitioned feeding behaviour among size classes within the Pomo Pit, where the smallest size class (TL < 120 mm P) feeds mainly on *E. krohni*, a typical zooplankton crustacean, whereas the food item with the highest frequency in the largest size classes was *M. poutassou*, confirming the known ontogenetic shift to a piscivorous diet^36^. The Bray–Curtis dietary dissimilarities suggested the presence of a high ontogenetic niche partitioning within the deep habitat samples confirming the ontogenetic dietary shifts for European hake that are well documented^36,37^. Nevertheless, the ontogenetic dietary differences were not emerging when looking at diet dissimilarities among size classes outside Pomo Pit and the partial dietary overlap among the five size classes of *M. merluccius* sampled in the shallower habitat confirms results presented previously in Riccioni et al.^35^. Since the Pomo pit is an area of aggregation for European hake^52^ and large individuals prey on smaller specimens, it is possible that the expression of ontogenetic trophic differences is also due to spatial segregation of size classes within Pomo pit for predator avoidance, that is less evident in the shallow area. Furthermore, it should be noted that the samples were collected in a very wide area outside Pomo pit that might comprise several environments resulting in a quite complex and variable diet for all size classes of this opportunistic predator. Our results suggested that small individuals have a partially opportunistic behaviour and a preference for highly energetic preys (like euphasiids and anchovies^53–56^). Indeed, nutritional conditions have been found to affect the swimming behaviour of juvenile fish^57,58^ and their body condition, as well as reduce their survival^59^.

### Consistency in niche partitioning over time, size-classes and space

The analyses highlighted a statistically significant dietary partition among the size classes collected in the two different habitats, and the variance detected in the data correlated perfectly with the depth variable, corroborating the presence of a niche separation linked to the presence/absence of some species at different bathymetries or to some peculiar metabolic demand of hakes in different habitats. This pattern of differentiation confirmed previous findings that at shallow depth the diet of European hake is not significantly different among size classes^35^, whereas remarkable and significant differences emerge when including specimens of depth > 200 m such as in Pomo Pit area. In particular, the high dissimilarity between the largest individuals sampled within the Pomo Pit (TL 250–299 P and TL ≥ 300 P) with all size classes sampled in the shallow area highlights the peculiarity of the Pomo Pit environment for the adult hakes.

The analysis of modularity lead to refine these findings through the identification of four compartments in the bipartite network, highlighting the presence of a restricted number of very frequent preys and a pool of rare preys. These four modules validated the differentiation signal detected in the samples collected within the Pomo Pit, clustering the smallest and largest size classes apart, and identifying further a higher interconnection between the two smallest size classes and among the largest size classes in the shallower samples. Moreover, the identified modules contained approximately the same prey species for the same size classes in samples collected in both 2016 and 2014. This stability in niche partitioning resulted in spite of the variation in species composition among the two years: in particular the extremely low number of *L. friesii* in 2016 with respect to 2014^35^. Although no information is available about the population dynamics of *L. friesii*, this result suggested an important temporal variation in the abundance of this species between the years 2014 and 2016. Overall, the comparison among data collected in two different years (2014 and 2016) provided evidence of a high degree of stability of the diet of European hake over time.

### Ecological and ecosystem considerations

The differentiation in feeding behaviour supported the presence of highly complex trophic relationships of *M. merluccius* in the NC Adriatic Sea ^35^ and the important role of the Pomo Pit as spawning/nursery area allowing access to specific food items exclusively or predominantly present in this habitat. The metabarcoding data indicate lower connectance and nestedness for European hake diet in the Pomo Pit than outside indicating larger number highly nested trophic interactions in the shallow waters than in the Pomo Pit. The two properties, connectance and nestedness, are correlated^60,61^ and their high values are providing to communities robustness and stability to disturbances through several mechanisms. For instance, high values for connectance and nestedness indicate that the predator have access to more scattered species, allowing it to compensate for eventual changes in the abundance of resources^62^. The lower value of the nestedness in the Pomo Pit than in shallow areas outside is coherent with a more specialized feeding of European hake within Pomo pit which is consistent with a less disturbed environment. Conversely, high connectance and nestedness indicates that individuals of all size classes have a high degree of niche overlap outside Pomo Pit to contrast disturbances and increase stability and resilience to perturbations through several trophic linkages. Lower connectance in less disturbed environments was also found before by comparing protected and exploited areas in the Adriatic Sea^63^ and thus our findings are coherent with the identification of relevant less disturbed habitat in Pomo Pit.

### Limitations and implications for ecosystem-based fisheries management

The complex trophic relationships detected in this study and the previous results^35^ are based on a higher efficiency of the DNA-based method in the taxonomic identification of prey in stomach content. Results emphasize the need to apply innovative scientific methods like the metabarcoding approach to study marine food webs and to integrate the current information about the feeding behaviour of marine organisms. However, it is relevant to consider that metabarcoding analyses provide a snapshot of the last meal, in particular for predatory fish species, which have relatively rapid gut throughput times, and differ from stable isotopes analyses which provide an information integrated over longer time span (a few months). In addition, metabarcoding cannot discern direct from secondary predations, i.e., the prey of the European hake food items. For instance, it cannot be excluded that some identified species were prey of *M. poutassou*, even if this species, inhabiting lower depth, is characterised by a narrower spectrum of prey^43^. Nevertheless, we did not observe the presence of zooplankton OTUs (which are the main prey for anchovies) or other species characteristic of detritivore’s diet, thus we can confidently expect that the very conservative bioinformatic approach applied in this study was filtering out the low quality sequences resulting from a higher degree of DNA degradation of the secondary predation prey. Metabarcoding cannot discern also the intra-species predation (cannibalism) which is known to occur for European hake^25,27,29,30^ and can affect some of the results obtained. Therefore, the integrated use of different methods (e.g., metabarcoding and stable isotopes) is recommended as it can provide an unprecedented insight into trophic relationships and give an important contribution to the implementation of an Ecosystem-Based Fisheries Management (EBFM). However, the metabarcoding approach highlighted the presence of several commercial species in the diet of European hake, including fish (e.g., *Engraulis encrasicolus*, *Sardina pilchardus, Mullus barbatus, Trachurus trachurus*) and invertebrates (e.g., *Illex coindetii, Eledone moschata, Parapenaeus longirostris*) of primary importance for Adriatic fisheries^12^. Therefore, a good representation of trophic links and feeding strategy for species such as European hake, that in the Adriatic is a voracious predator of other commercial species, could ease the implementation of EBFM. The disentanglement of complex trophic relationships, in facts, could allow predicting trophic cascades of exploitation of apex predators^6^, accounting for indirect fisheries interactions^64,65^ and include trade-offs in managing mixed-fisheries^66^.

In this context our results contribute with several important aspects to the fine description of the trophic relationships in the ecosystem of Pomo Pit: although the module analysis showed that the predatory behaviour is the same within and outside the Pomo Pit, the metabarcoding approach highlighted differences in the prey detected. In particular, the high prevalence of *E. encrasicolus* in the diet suggests its extreme importance for the trophism of *M. merluccius*. Our reconstruction of trophic habits of *M. merluccius* in the Northern Adriatic Sea, highlighted the essential role of the Pomo Pit area for the almost exclusive presence of some prey species. In this area is present a favourable habitat for the development of European hake and the peculiar ontogenetic shift revealed in this deep area of the Northern Adriatic confirmed the uniqueness of this ecosystem for species connectivity and food webs. Moreover, the species detected in the stomachs and the selectivity index, revealed a feeding preference for several species inhabiting this area, in particular for the youngest class, strengthening the hypothesis of the presence of an EFH^67^, namely an habitat “necessary to fish for spawning, breeding, feeding or growth to maturity”^68^. These areas are characterised by important ecological functions and are sensitive to environmental degradation.

## Methods

### Sampling

*Merluccius merluccius* is a commercial species therefore neither special permits nor ethics approval were required for their sampling, all methods were carried out in accordance with relevant guidelines and regulations. One hundred European hake specimens were collected in 2016, from 46 to 260 m depth in the Northern Adriatic Sea from the Gulf of Trieste to Pomo Pit (Fig. 1 and Supplementary Table S5) within the framework of International Bottom Trawl Survey in the Mediterranean (MEDITS, MEDITS-Handbook, v.7, 2013) during the late summer season (August and September). Additional samples (100) collected in 2014^35^ along the Italian coasts of the Northern Adriatic Sea were further processed. Specimens were categorized according to five size classes previously identified on the basis of size distribution by bathymetric and geographical strata, abundance and feeding habits^35^: TL < 120 mm, (within the Pomo Pit) and TL 120–149 mm (outside the Pomo Pit); TL 150–199 mm; TL 200–249 mm; TL 250–299 mm; TL ≥ 300 mm. Ten individuals per size class from the two different habitats, i.e., shallow water outside the Pomo Pit (S) and deep water within the Pomo Pit (P; ≤ 150 m and > 150 m depth, respectively), were selected for the metabarcoding analyses. The stomachs were dissected *post-mortem* and preserved in 95% ethanol at − 20 °C until metabarcoding analyses. To avoid cross-contamination, dissection tools were flame sterilized between individuals and lab surfaces were decontaminated with bleach.

### Molecular analyses

Stomach contents over 100 µL were homogenised separately with a blender; a 100 µL subsample of the homogenate was used for the molecular analyses. All genomic DNAs were extracted by using a commercial kit (DNeasy Blood & Tissue Kit, QIAGEN). Samples were processed in small batches representing five size classes of *M. merluccius* (10 specimens each) with an extraction blank to monitor for potential cross-contamination in a separate room designated to conduct molecular diet analyses. PCR amplification was performed in two replicates in a total volume of 25 μL with 0.75 μL of 10 μM of each forward and reverse primers (mlCOIintF and jgHCO2198^33^, mlCOIintF—GGWACWGGWTGAACWGTWTAYCCYCC, jgHCO2198 – TAIACYTCIGGRTGICCRAARAAYCA), 0.2 μL of AmpliTaq Gold® DNA Polymerase (ThermoFisher) 5 UμL^-1^, 2 μL of 25 mM Mg2 + , 0.5 μL of 10 mM dNTP, 1 mg/mL BSA and 3 μL of genomic DNA. We used a “touchdown” PCR profile^35^ to minimize the probability of non-specific amplifications. We carried out 16 initial cycles: denaturation for 10 s at 95 °C, annealing for 30 s at 62 °C (− 1 °C per cycle) and extension for 60 s at 72 °C, followed by 25 cycles at 46 °C annealing temperature (− 0.2 °C per cycle). We included no-template controls in all PCRs and the products were checked on 1.5% agarose gels. We used twenty tagged primers for DNA amplification and library preparation. All the tagged amplicons (313 bp plus tag) were purified with Sera-Mag SpeedBeads (GE Healthcare Life Sciences) purification protocol^69^, quantified with Qubit fluorometer and pooled in equimolar concentration. Approximately 18 × 10^6^ paired-end sequences were obtained from Illumina MiSeq sequencing (2 × 250), performed by Fasteris SA (Fasteris SA, 1228 Plan-les-Ouates, Switzerland) following Metafast protocol (PCR-free protocol for library preparation, https://www.fasteris.com/dna/?q=content/metafast-protocol-amplicon-metagenomic-analysis).

### Bioinformatic and statistical analyses

Sequence demultiplexing, quality control, PCR and sequencing error filtering were performed using OBITools software (Boyer et al., 2016; http://metabarcoding.org/obitools/doc/welcome.html; last access: February 2019). The *illuminapairedend* script was used to perform a micro-assembly of paired-end reads. Sequences with Illumina fastq quality scores < 30 across the head, tail or total length of the sequence were discarded. We used *ngsfilter* script to assign the reads to each sample through barcode identification (11 × 10^6^ sequences). Only the sequences longer than 100 bp were retained and dereplicated by using *obiuniq* script. We further filtered the sequences and those with count < 10 were discarded, moreover the *obiclean* script were used to detect the potential PCR errors selecting only sequences with the ‘*head*’ status and abundance higher than 0.05%.

Two different approaches have been used to evaluate the diet composition of *M. merluccius* from the metabarcoding data: a) sequence relative occurrence (i.e., presence/absence), b) OTUs (Operational Taxonomic Units) Relative Abundance (ORA), which is the proportion of unique OTUs in a sample divided by the final number of OTUs (after bioinformatic processing) in that sample. We used ORA data to compute the Jacobs’ index (see below).

The OTUs’ assignment was performed by BLASTn^71^ searches of OTU representative sequences against full GenBank database (February 2019). We used BLAST algorithm optimized for very similar sequences (megablast) on the nucleotide collection (nr/nt) that includes all GenBank + EMBL + DDBJ + PDB sequences restricting the search to sequences with > 95% of similarity and considering the top five hits for each OTU. Moreover, we assigned an OTU at the species level when similarity to the reference barcode was ≥ 97% and when there was no ambiguity in the taxonomic assignment of OTUs (within the top five hits with the same similarity percentage only sequences belonging to the same species appeared^35^). All the OTUs which did not match these rules were not assigned, we considered only sequences assigned at the species level. No bacterial and human OTUs were detected with a similarity match > 95%, however 25% of OTUs could not be assigned (NA’s) and 30% of all the OTUs detected a match with sequences of *M. merluccius*.

Inter-size class variability was measured using Bray–Curtis dissimilarities^72^, which range from 0 (complete overlap) to 1 (complete nonoverlap), to compute pairwise community distance matrices and examine differences in beta diversity. Occurrence data were standardised by Hellinger transformation^73^ and dissimilarities between all pairs of different size classes as well as mean within size classes were calculated using Bray–Curtis dissimilarity coefficient. Pairwise differences in intra-classes dietary variation (i.e., dispersion) were tested using the functions *betadisper* and *permutest* with 1000 permutations and bias adjustment. Patterns of sample dissimilarity were visualized using PCoA. A non-parametric analysis of similarity (R-vegan function *anosim*; 1,000 Monte Carlo permutations) was used to test the null hypothesis of no difference in species composition among size classes.

Correlation analyses with environmental variables were carried out by using the *vegan* package^72^ in R^74^. The function *envfit* (Family: gaussian, 1,000 permutations) was used to correlate depth with the ordination obtained for the two P and S habitats and to obtain and plot the corresponding gradient vector in PCoA ordination. Similarly, depth values were fitted as smooth surfaces created using a generalized additive model via the *ordisurf* function (Family: gaussian, 1,000 permutations). To refine this analysis, we performed a permutational PERMANOVA test that can accommodate both categorical and continuous predictor variables (R-vegan function *adonis*; 1,000 permutations) considering both size classes and habitat as independent categorical factor and testing also for the interaction between these two factors. Sample-based species accumulation curves were computed by using the *specaccum* function using the “rarefaction” method to compare community diversity between the two habitats.

The R package *bipartite*^75^ was used to build food web networks, to create a heat map for the visualisation of bipartite graph and for modules identification within the networks (*metaComputemodules* function with default values and n = 100, it applies Newman’s modularity measure in a bipartite weighted version to a bipartite graph to identify subcommunities connections). Several indices such as degree which is the number of links per species, weighted NODF (a nestedness metric based on overlap and decreasing fill, incorporating also information on the weights of the link^76^) which measures the presence of substructures and in this case is related to niche overlap, connectance which is the realised proportion of possible links, were computed by using the function *networklevel* considering the data separately for the two different Pomo Pit (P) and Shallow (S) habitats.

In addition, interannual variation (2014 vs 2016) of feeding behaviour of Adriatic European hake was tested by performing a *bipartite* analysis using also previously obtained stomach content metabarcoding data^35^. Dietary selectivity and preference were assessed with the function *selectapref* and by computing the Jacobs’ index^77^ using MEDITS density index “number of individuals/km^2^” as a proxy for prey availability. In this analysis, for prey availability we considered the same hauls from which *M*. *merluccius* stomachs were collected, and we used OTUs Relative Abundance (ORA) data, the proportion of unique OTUs in a sample divided by the final number of OTUs (after bioinformatic processing) in that sample. For all the analyses we used R version 3.5.2^74^.

## Supplementary Information

Supplementary Information.

## Data Availability

Illumina DNA sequences obtained during the current study were deposited in the European Nucleotide Archive (ENA, http://www.ebi.ac.uk/ena) under the accession number PRJEB34163 (http://www.ebi.ac.uk/ena/data/view/PRJEB34163).
