# Supplementary material for: DNA metabarcoding suggests dietary niche partitioning in the Adriatic European hake
Source: Sci Rep. 2022 Jan 25;12:1343. doi: 10.1038/s41598-022-05346-0 (PMC8789918; doi:10.1038/s41598-022-05346-0)
Supplement: Supplementary file 1 — Supplementary Information. [file 41598_2022_5346_MOESM1_ESM.docx]

**Supplemental Information for:**

**DNA metabarcoding suggests dietary niche partitioning in the Adriatic European hake**

Giulia Riccioni, Marco Stagioni, Chiara Manfredi, Fausto Tinti, Corrado Piccinetti, Simone Libralato

**Table S1.** List of species detected in previous studies performed on European hake diet in the Mediterranean Sea.

| Vertical distribution and feeding patterns of the juvenile European hake, *Merluccius merluccius* in the NW Mediterranean | | | |  |  |  |  |  |  |
| --- | --- | --- | --- | --- | --- | --- | --- | --- | --- |
| Anna Bozzano, Francesco Sardà, José Rı́os | | | |  |  |  |  |  |  |
| Fisheries Research 73 (2005) 29–36 | | | |  |  |  |  |  |  |
| **Region** Catalan coast (north-western Mediterranean) | | | | | | |  |  |  |
| **Hake size** Juveniles | | | | | | |  |  |  |
|  |  | | | | | | |  |  |
| **Cephalopoda** | **Mysidacea** | **Osteychthyes** |  |  |  |  |  |  |  |
| *Sepia orbignyana* | *Lophogater typicus* | *Gadiculus argenteus* |  |  |  |  |  |  |  |
| *Alloteuthis media* | *Anchialina agilis* | *Maurolicus muelleri* |  |  |  |  |  |  |  |
| Sepiolidae | *Gastrosaccus sp.* | Myctophidae |  |  |  |  |  |  |  |
| **Copepoda** | *Mysidacea* unid. | *Deltentosteus quadrimaculatus* |  |  |  |  |  |  |  |
| Copepoda unid. | **Crustacea decapoda** | *Lesueurigobius friesii* |  |  |  |  |  |  |  |
| **Isopoda** | *Chlorotocus crassicornis* | *Callionymus maculatus* |  |  |  |  |  |  |  |
| *Gnathia sp.* | *Solenocera membranacea* | *Antonogadus megalokynodon* |  |  |  |  |  |  |  |
| Isopoda unid. | *Processa nouveli* | Paralepididae |  |  |  |  |  |  |  |
| **Amphipoda** | *Pasiphaea sivado* | Osteichthyes unid |  |  |  |  |  |  |  |
| Amphipoda unid. | *Philocheras sculptus* |  |  |  |  |  |  |  |  |
| **Euphausiacea** | *Brachyura* |  |  |  |  |  |  |  |  |
| *Euphasia krohnii* | *Scyllarus arctus* |  |  |  |  |  |  |  |  |
| *Nyctiphanes couchii* | *Scyllarus posteli* |  |  |  |  |  |  |  |  |
| *Euphausiacea* unid. | Crustacea Decapoda unid. |  |  |  |  |  |  |  |  |
|  | Crustacea unid. |  |  |  |  |  |  |  |  |
|  |  |  | | | | | | |  |
| Diet of the european hake *Merluccius merluccius* (Pisces: Merlucciidae) in the Western Mediterranean (Gulf of Lions) | | | | |  |  |  |  |  |
| A. Bozzano, L. Recasens, P. Sartor | | | | |  |  |  |  |  |
| Scientia Marina 61 (1) 1997 1–8 | | | | |  |  |  |  |  |
| **Region** Gulf of Lions | | | | | |  |  |  |  |
| **Hake size** Juveniles – Adults | | | | | |  |  |  |  |
|  | | | | | |  |  |  |  |
| **Cephalopoda** | **DECAPODA** | **PISCES** | | | | | | |  |
| *Sepia orbignyvana* | *Aristeus antennatues* | *Antonogadus megalokynodon* | | | | | | |  |
| *Sepietta oweniana* | *Solenocera membranacea* | *Gadiculus argenteus* | | | | | | |  |
| Unid. Sepiolidae | *Sergestes arcticus* | *Phycis blennoides* | | | | | | |  |
| *Alloteuthis sp.* | *Alpheus glaber* | *Trisopterus m. capelanis* | | | | | | |  |
| *Todarodes sagittatus* | *Chlorotocus crassicornis* | *Micromesistius poutassou* | | | | | | |  |
| Unid. Ommastrephidac | *Plesionika sp.* | *Mora moro* | | | | | | |  |
| Unid. Cephalopoda | *Pontocaris lacazei* | *Merluccius merluccius* | | | | | | |  |
| **MYSIDACEA** | *Pontocaris cataphracta* | *Lesueurigobius friesii* | | | | | | |  |
| *Lophogaster typicus* | *Pontocaris sp.* | *Deltentosteus quadrimaculdatus* | | | | | | |  |
| *Siriella sp.* | *Pontophilus spinosus* | *Cristallogobius linearis* | | | | | | |  |
| Unid. Mysidacea | Unid. Crangonidae | Unid. Gobidae | | | | | | |  |
| **ISOPODA** | *Pasiphaea sivado* | Unid. Myctophidae | | | | | | |  |
| Unid. Cymothoidea | *Processa canaliculata* | *Callionymus maculatus* | | | | | | |  |
| **AMPHIPODA** | Unid. Decapoda | *Cepola rubescens* | | | | | | |  |
| *Phronima sedentaria* | *Processa sp.* | *Echiodon dentatus* | | | | | | |  |
| *Vibilia armata* | *Callianassa sp.* | Unid. Congridae | | | | | | |  |
| Unid. Lysianassidae | *Upogebia sp.* | *Engraulis encrasicolus* | | | | | | |  |
| Unid. Gammaridae | Unid. Crustacca | *Sardina pilchardus* | | | | | | |  |
| **EUPHAUSIACEA** | **CRINOIDEA** | *Scomber sp.* | | | | | | |  |
| *Meganyctiphanes norvegica* | *Leptomerra phalangium* | Unid. Osteichthyes | | | | | | |  |
| Unid. Euphausiacea | **THALIACEA** |  | | | | | | |  |
|  | Unid. Thaliacea |  | | | | | | |  |
|  |  |  | | | | | | |  |
|  |  |  | | | | | | | |

| Feeding habits of European hake (*Merluccius merluccius*) in the central Mediterranean Sea | | |  |  |  |
| --- | --- | --- | --- | --- | --- |
| Paolo Carpentieri, Francesco Colloca, Massimiliano Cardinale, Andrea Belluscio, Giandomenico D. Ardizzone | | |  |  |  |
| Fish. Bull. 103:411–416 (2005) | | |  |  |  |
| **Region** Central western coasts of Italy | | |  |  |  |
| **Hake size** Juveniles – Adults | | |  |  |  |
| **Cephalopoda** | **Pisces** |  |  |  |  |
| *Alloteuthis media* | *Argentina sphyraena* |  |  |  |  |
| *Sepietta oweniana* | *Arnoglossus laterna* |  |  |  |  |
| Unid. Sepiolidae | *Arnoglossus sp.* |  |  |  |  |
| Unid. Cephalopoda | *Callionymus sp.* |  |  |  |  |
| **Crustacea** | Centracanthidae |  |  |  |  |
| *Alpheus glaber* | *Centracanthus cirrus* |  |  |  |  |
| Aristeidae | *Clorophthalmus agassizi* |  |  |  |  |
| *Aristeus antennatus* | *Conger conger* |  |  |  |  |
| *Chlorotocus crassicornis* | *Echiodon dentatus* |  |  |  |  |
| Crangonidae | *Engraulis encrasicolus* |  |  |  |  |
| Pandalidae | *Gadiculus argenteus* |  |  |  |  |
| *Parapenaeus longirostris* | Gobiidae |  |  |  |  |
| *Pasiphaea multidentata* | *Gobius quadrimaculatus* |  |  |  |  |
| *Pasiphaea sivado* | *Lepidotrigla dieuzedei* |  |  |  |  |
| *Plesionika heterocarpus* | *Lesuerigobius friesii* |  |  |  |  |
| *Plesionika sp.* | *Merluccius merluccius* |  |  |  |  |
| *Pontocaris lacazei* | *Mullus barbatus* |  |  |  |  |
| *Pontophilus spinosus* | Myctophidae |  |  |  |  |
| *Processa sp.* | *Nettastoma melanurum* |  |  |  |  |
| *Solenocera membranaca* | *Sardina pilchardus* |  |  |  |  |
| *Squilla sp.* | *Sphyraena sphyraena* |  |  |  |  |
| Unid. Decapoda | *Spicara flexuosa* |  |  |  |  |
| *Lophogaster typicus* | *Spicara sp.* |  |  |  |  |
| *Nictiphanes couchi* | *Trachurus trachurus* |  |  |  |  |
| Unid. Euphasiacea | *Trisopterus m. capelanus* |  |  |  |  |
| Unid. Isopoda | Unid. Osteichthyes |  |  |  |  |
|  | *Raja sp.* |  |  |  |  |
| Prey preferences and ontogenetic diet shift of European hake *Merluccius merluccius* (Linnaeus, 1758) in the central | | | |  |  |
| Virginia Carrozzi, Manfredi Di Lorenzo, Daniela Massi, Antonino Titone, Giandomenico Ardizzone, Francesco Colloca | | | |  |  |
| Regional Studies in Marine Science 25 (2019) 100440 | | | |  |  |
| **Region** North sector of the Strait of Sicily, central Mediterranean Sea | | | | |  |
| **Hake size** Juveniles – Adults | | | | |  |
|  | | | | |  |
| **Cephalopoda** | **Crustacea** | **Pisces** | | | |
| *Alloteuthis sp.* | *Parapenaeus longirostris* | *Trachurus trachurus* | | | |
| *Illex coindetii* | *Chlorotocus crassicornis* | *Merluccius merluccius* | | | |
| *Sepia officinalis* | *Alpheus glaber* | *Lepidopus caudatus* | | | |
| *Loligo vulgaris* | *Aristaeomorpha foliacea* | *Engraulis encrasicolus* | | | |
| *Sepietta oweniana* | *Plesionika acanthonotus* | *Sardina pilchardus* | | | |
| Unid. Cephalopoda | *Processa acutirostris* | *Argentina sphyraena* | | | |
|  | *Processa edulis* | *Lepidotrigla cavillone* | | | |
|  | *Nephrops norvegicus* | *Citharus linguatula* | | | |
|  | Munida iris | *Ceratoscopelus maderensis* | | | |
|  | *Goneplax rhomboides* | *Lampanyctus crocodilus* | | | |
|  | *Pasiphaea sivado* | *Gaidropsarus biscayensis* | | | |
|  | *Scyllarus arctus* | *Gadiculus argenteus* | | | |
|  | *Euphasia krohnii* | *Solea sp.* | | | |
|  | *Nyctiphanes couchi* | *Cepola macrophthalma* | | | |
|  | *Lophogaster typicus* | *Phycis blennoides* | | | |
|  | Unid. Euphausiaea | Gobiidae | | | |
|  | Unid. Decapoda | *Lesueurigobius friesii* | | | |
|  | Unid. Crustacea | *Macroramphosus scolopax* | | | |
|  |  | Unid. Osteychthyes | | | |

|  | | | |  |
| --- | --- | --- | --- | --- |
| Infuence of environmental variables on the feeding and diet of European hake (*Merluccius merluccius*) on the Mediterranean Iberian coasts | | |  |  |
| J.E. Cartes P, J. Rey O, D. Lloris and L. Gil de Sola | | |  |  |
| J. Mar. Biol. Ass. U.K. (2004), 84, 831-835 | | |  |  |
| **Region** Coasts of the Iberian Peninsula (Western Mediterranean) | | |  |  |
| **Hake size** | | |  |  |
|  |  |  | | |
| **Main prey** | **Minor prey** |  |  |  |
| *Sardinella aurita* | *Lampanyctus crocodilus* |  |  |  |
| *Sardina pilchardus* | *Notolepis rissoi* |  |  |  |
| *Trachurus trachurus* | *Argentina sphyraena* |  |  |  |
| *Boops boops* | *Micromesistius poutassou* |  |  |  |
| Myctophidae | *Gaidropsarus megalokynodon* |  |  |  |
| *Lepidopus caudatus* | *Gadiculus argenteus* |  |  |  |
| *Merluccius merluccius* | *Aphia minuta* |  |  |  |
| *Phycis blennoides* | *Spicara sp.* |  |  |  |
| Osteychthyes unident. | *Cepola rubescens* |  |  |  |
| Euphausiacea | *Solenocera membranacea* |  |  |  |
| Mysids | *Pasiphaea sivado* |  |  |  |
| Gammaridean Amphipods | *Sergestes arcticus* |  |  |  |
| Decapoda Natantia unident. | *Plesionika gigliolii* |  |  |  |
| *Chlorotocus crassicornis* | *Processa sp.* |  |  |  |
| *Plesionika heterocarpus* | *Pontocaris lacazei* |  |  |  |
| *Alpheus glaber* | *Liocarcinus depurator* |  |  |  |
| Crustacea unident. | *Lophogaster typicus* |  |  |  |
|  | *Alloteuthis media* |  |  |  |
|  | Sepiolidae. |  |  |  |

| Spatio-temporal variation in diet may affect condition and abundance of juvenile European hake in the Gulf of Lions (NW Mediterranean) | | |  |  |
| --- | --- | --- | --- | --- |
| Franck Ferraton, Mireille Harmelin-Vivien, Capucine Mellon-Duval, Arnaud Souplet | | |  |  |
| Mar Ecol Prog Ser **Vol. 337: 197–208, 2007** | | |  |  |
| **Region** Gulf of Lions (NW Mediterranean) | | |  |  |
| **Hake size** Juveniles | | |  |  |
|  | | | |  |
| **Crustaceans** |  |  | | |
| Amphipods |  |  | | |
| Euphausiids |  |  | | |
| Mysids |  |  | | |
| Isopods |  |  | | |
| Cumaceans |  |  | | |
| Natantids |  |  | | |
| Macrurans |  |  | | |
| Unidentified crustaceans |  |  | | |
| **Fishes** |  |  | | |
| Benthic fishes |  |  | | |
| Demersal fishes |  |  | | |
| Pelagic fishes |  |  | | |
| Unidentified fishes |  |  | | |
| **Cephalopods** |  |  | | |
| **Polychaetes** |  |  | | |

| Trophic ecology of the European hake in the Gulf of Lions, north western Mediterranean Sea | | |  |  |
| --- | --- | --- | --- | --- |
| Capucine Mellon-Duval, Mireille Harmelin-Vivien, Luisa Métral, Véronique Loizeau, Serge Mortreux, David Roos, Jean-Marc Fromentin. | | |  |  |
| SCI. MAR. 81(1), March 2017, 7-18. ISSN-L 0214-8358 doi: http://dx.doi.org/10.3989/scimar.04356.01A | | |  |  |
| **Region** Gulf of Lions | | |  |  |
| **Hake size** Juveniles – Adults | | | |  |
| **CEPHALOPODS** | **CRUSTACEANS** | **FISH** | | |
| *Alloteuthis sp.* | *Philocheras sp.* | Demersal fish | | |
| *Sepiola sp.* | *Plesionika sp.* | *Argentina sphyraena* | | |
| Teuthida Unid. | *Processa canaliculata* | Capros aper | | |
| Cephalopods Unid. | *Processa sp.* | *Cepola macrophthalma* | | |
|  | Processidae Unid. | *Epigonus denticulatus* | | |
| **CRUSTACEANS** | *Solenocera membranacea* | *Gaidropsaurus biscayensis* | | |
| Amphipoda | Solenoceridae Unid. | *Merluccius merluccius* | | |
| *Ampelisca sp.* | Reptantia | *Trisopterus minutus* | | |
| *Vibilia armata* | *Liocarcinus depurator* | Pelagic fish | | |
| *Vibilia sp.* | Suprabenthos Unid. | *Engraulis encrasicolus* | | |
| Amphipoda Unid. | Crustacea Unid. | *Micromesistius poutassou* | | |
| Cumacea |  | *Sardina pilchardus* | | |
| Cumacea Unid. | **FISH** | *Scomber scombrus* | | |
| Euphausiacea | Benthic fish | *Sprattus sprattus* | | |
| Euphausiacea Unid. | Callionymidae Unid. | *Trachurus mediterraneus* | | |
| Mysidacea | *Conger conger* | *Trachurus sp.* | | |
| *Leptomysis sp.* | Deltentosteus quadrimaculatus | Pelagic fish Unid. | | |
| Mysidacea Unid. | Gobidae Unid. | Fish Unid. | | |
| Natantia | *Gobius niger* |  | | |
| Alpheidae Unid. | *Lepidopus caudatus* |  | | |
| *Alpheus glaber* | *Lepidotrigla cavillone* |  | | |
| *Alpheus sp.* | *Lesueurigobius friesii* |  | | |
| Caridea Unid. | *Mullus sp.* |  | | |
| Natantia Unid. | *Phycis blennoides* |  | | |
| *Pasiphaea sivado* | *Phycis sp.* |  | | |
| Penaeidae Unid. | Triglidae Unid. |  | | |

| Feeding Habits of European Hake, *Merluccius merluccius* (Actinopterygii: Gadiformes: from the Northeastern Mediterranean Sea Merlucciidae) | | |  |  |
| --- | --- | --- | --- | --- |
| Marco STAGIONI, Stefano MONTANINI, and Maria VALLISNERI | | |  |  |
| ACTA ICHTHYOLOGICA ET PISCATORIA (2011) 41 (4): 277–284 | | |  |  |
| **Region** Adriatic Sea | | |  |  |
| **Hake size** Juveniles – Adults | | |  |  |
|  | | | |  |
| **Cephalopoda** | **Actinopterygii** |  | | |
| **Gastropoda** | *Engraulis encrasicolus* |  | | |
| **Bivalvia** | *Cepola macrophthalma* |  | | |
|  | *Gaidropsarus biscayensis* |  | | |
| **Crustacea** | *Gobius niger* |  | | |
| *Processa sp.* | *Trisopterus minutus capelanus* |  | | |
| *Philocheras sp.* | *Merlangius merlangus* |  | | |
| *Alpheus glaber* | *Gadiculus argenteus* |  | | |
| *Solenocera membranacea* | *Merluccius merluccius* |  | | |
| *Philocheras bispinosus* | *Lesuerigobius friesii* |  | | |
| *Lophogaster typicus* | *Micromesistius poutassou* |  | | |
| *Chlorotocus crassicornis* | *Callionymus sp.* |  | | |
| *Processa macrophthalma* | *Callionymus maculatus* |  | | |
| *Rissoides desmaresti* | *Gaidropsarus sp.* |  | | |
| *Liocarcinus sp.* | *Argentina sphyraena* |  | | |
|  | *Maurolicus muelleri* |  | | |
|  | *Sardina pilchardus* |  | | |
|  | *Atherina boyeri* |  | | |

| Daily ration and feeding activity of juvenile hake in the central Mediterranean Sea | | |  |  |
| --- | --- | --- | --- | --- |
| P. Carpentieri, F. Colloca and G. Ardizzone | | |  |  |
| Journal of the Marine Biological Association of the United Kingdom, 2008, 88(7), 1493–1501 | | |  |  |
| **Region** Western coast of central Italy (central Mediterranean Sea) | | |  |  |
| **Hake size** Juveniles | | |  |  |
|  | | | |  |
| **Crustacea** |  |  | | |
| *Nyctiphanes couchii* |  |  | | |
| Unid. Euphasiacea |  |  | | |
| *Apheus glaber* |  |  | | |
| *Clorotocus crassicornis* |  |  | | |
| *Parapenaeus longirostris* |  |  | | |
| *Pasiphaea sivado* |  |  | | |
| *Plesionika heterocarpus* |  |  | | |
| *Pontophilus spinosus* |  |  | | |
| *Processa canaliculata* |  |  | | |
| *Solenocera membranacea* |  |  | | |
| Unid. Decapoda |  |  | | |
| **Cephalopoda** |  |  | | |
| *Alloteuthis media* |  |  | | |
| *Sepietta oweniana* |  |  | | |
| Unid. Sepiolidae |  |  | | |
| **Teleostea** |  |  | | |
| Gobidae |  |  | | |
| *Engraulis encrasicolus* |  |  | | |
| Unid. Osteichtyes |  |  | | |

| Mesoscale variability in the trophic ecology of the European hake *Merluccius merluccius* in the Strait of Sicily | | |  |
| --- | --- | --- | --- |
| E. Fanelli, P. Rumolo, M. Barra, G. Basilone, S. Genovese, A. Bonann | | |  |
| Hydrobiologia (2018) 821:57–72 | | |  |
| **Region** Strait of Sicily (central Mediterranean Sea) | | |  |
| **Hake size** Juveniles – Adults | | |  |
|  | | |  |
| **MOLLUSCA** | **ARTHROPODA** | **ECHINODERMATA** | |
| Bivalvia | Crustacea | *Elpidia glacialis* | |
| Bivalvia unid. | Mysidacea | Holothuroidea unid. | |
| Gasteropoda | *Erytrops* sp. | Echinodermata unid. | |
| *Bittium* sp. | *Leptomysis gracilis* | FORAMINIFERA | |
| Gasteropoda unid. | *Leptomysis mediterranea* | Benthic foraminiferans | |
| Cephalopoda | *Lophogaster typicus* |  | |
| *Allotheutis media* | *Siriella jaltensis* |  | |
| *Allotheutis sp.* | Mysidacea unid. |  | |
| *Neorossia caroli* | Euphausiacea |  | |
| *Rondelentiola minor* | *Nyctiphanes couchii* |  | |
| *Sepietta neglecta* | Euphausiacea unid. |  | |
| *Sepietta oweniana* | Stomatopoda |  | |
| Sepiolidae unid. | *Parasquilla ferussaci* |  | |
| Cephalopoda unid. | Decapoda |  | |
| **POLYCHAETA** | *Alpheus glaber* |  | |
| Polychaeta unid. | *Chlorotocus crassicornis* |  | |
|  | *Philocheras bispinosus* |  | |
|  | *Philocheras trispinosus* |  | |
|  | Decapoda unid. |  | |
|  | Crustacea unid. |  | |
|  |  |  | |

| Assessing the effects of a trawling ban on diet and trophic level of hake, *Merluccius merluccius*, in the southern Tyrrhenian Sea | | |  |  |
| --- | --- | --- | --- | --- |
| MAURO SINOPOLI, EMANUELA FANELLI, GIOVANNI D’ANNA, FABIO BADALAMENTI and CARLO PIPITONE | | |  |  |
| SCI. MAR., 76(4), December 2012, 677-690. ISSN 0214-8358 doi: 10.3989/scimar.03564.29A | | |  |  |
| **Region** Northern Sicily (Western Mediterranean) | | |  |  |
| **Hake size** Juveniles – Adults | | |  |  |
|  | | | |  |
| **Cephalopods** | **Fish** |  | | |
| *Sepietta sp* | *Antonogadus megalokydon* |  | | |
|  | *Cepola macrophthalma* |  | | |
| **Crustaceans** | *Deltentosteus quadrimaculatus* |  | | |
| *Alpheus glaber* | *Gadiculus argenteus* |  | | |
| *Parapenaeus longirostris* | Gadide |  | | |
| *Scyllarus* cfr *arctus* | *Gobius niger* |  | | |
| *Solenocera membranacea* | *Merluccius merluccius* |  | | |
| *Pontocaris lacazei* | *Mullus barbatus* |  | | |
| *Processa sp.* | Scorpaenidae |  | | |
| *Rocinela dumerili* | Triglidae |  | | |
| Euphausiacea | *Lesueurigobius suerii* |  | | |
| Decapoda Larvae | *Argentina sphyraena* |  | | |
| *Lophogaster typicus* | *Sardina pilchardus* |  | | |
| Mysidacea | *Engraulis encrasicolus* |  | | |
| *Ampelisca typica* | *Paralepis speciosa* |  | | |
| *Dyastilis* sp. | *Sardinella aurita* |  | | |
| *Monoculodes* sp. | *Spicara sp.* |  | | |
|  | *Trachurus trachurus* |  | | |
|  | *Lepidopus caudatus* |  | | |
|  | Osteichthyes larvae |  | | |

**Table S2**. Abundance of prey species (number of specimens per species) in the 10 NC Adriatic samples of *M. merluccius*, identified for each size class/habitat (combination of size class and habitat; P: sample collected within the Pomo Pit; S: individuals collected outside the Pomo Pit).

|  | **< 120 P** | **150-199 P** | **200-249 P** | **250-299 P** | **≥ 300 P** | **120-149 S** | **150-199 S** | **200-249 S** | **250-299 S** | **≥ 300 S** |
| --- | --- | --- | --- | --- | --- | --- | --- | --- | --- | --- |
| **Identified species** | **sample_1** | **sample_2** | **sample_3** | **sample_4** | **sample_5** | **sample_6** | **sample_7** | **sample_8** | **sample_9** | **sample_10** |
| *Alpheus glaber* |  |  | 1 |  |  | 4 | 1 | 1 | 3 | 1 |
| *Arnoglossus laterna* |  |  |  |  |  | 1 |  |  |  |  |
| *Chelidonichthys cuculus* |  | 1 |  |  |  |  |  |  |  |  |
| *Chlorotocus crassicornis* |  |  | 1 |  |  |  | 2 |  |  |  |
| *Eledone moschata* | 1 |  |  |  |  | 1 |  |  |  |  |
| *Engraulis encrasicolus* | 3 | 5 | 5 | 2 | 1 | 4 | 3 | 3 | 6 | 3 |
| *Euphausia krohni* | 3 |  |  |  |  |  |  |  |  |  |
| *Gadiculus argenteus* |  |  |  |  | 1 |  |  |  |  |  |
| *Gaidropsarus macrophthalmus* |  |  |  |  |  | 1 |  |  |  |  |
| *Gracilechinus acutus* |  |  |  |  |  | 1 |  |  |  |  |
| *Halice walkeri* |  |  |  |  |  | 1 |  |  |  |  |
| *Illex coindetii* |  |  |  | 1 |  |  |  |  |  |  |
| *Lesueurigobius friesii* |  |  |  |  |  | 1 | 1 |  |  |  |
| *Maurolicus muelleri* | 1 |  |  | 1 |  |  |  |  |  |  |
| *Meganyctiphanes norvegica* |  |  |  | 1 |  |  |  |  |  |  |
| *Merlangius merlangus* |  |  |  | 1 |  |  |  |  |  |  |
| *Micromesistius poutassou* |  |  | 5 | 3 | 9 |  |  |  |  | 1 |
| *Mullus barbatus* |  |  |  |  |  | 1 |  | 1 | 3 | 1 |
| *Nematoscelis megalops* | 1 |  | 1 |  |  |  |  |  |  |  |
| *Parapenaeus longirostris* |  |  |  | 2 | 1 |  |  |  |  |  |
| *Philocheras bispinosus* |  |  |  |  |  | 1 |  |  |  |  |
| *Processa nouveli* |  |  |  |  |  | 6 | 2 | 3 | 1 | 1 |
| *Raja miraletus* |  |  |  |  |  | 1 |  |  |  | 1 |
| *Rondeletiola minor* |  |  | 1 |  |  |  |  |  |  |  |
| *Sardina pilchardus* |  |  |  |  |  |  |  | 1 | 2 | 3 |
| *Scorpaena notata* |  |  |  |  |  | 1 |  |  |  | 1 |
| *Serranus hepatus* |  |  |  |  |  | 2 |  |  |  |  |
| *Solenocera membranacea* |  |  |  |  |  | 3 | 1 | 1 | 2 | 2 |
| *Todarodes sagittatus* |  |  |  |  | 1 |  |  |  |  |  |
| *Todaropsis eblanae* |  |  |  | 1 | 1 |  |  |  |  |  |
| *Trachurus trachurus* |  |  | 1 | 1 |  | 1 |  |  |  |  |
| *Trisopterus capelanus* |  |  | 1 |  |  |  |  |  |  |  |

**Table S3**. Mean within-size class Bray-Curtis dissimilarity coefficient computed in the 10 NC Adriatic gropus of *M. merluccius*, identified for each size class/habitat (combination of size class and habitat; P: sample collected within the Pomo Pit; S: individuals collected outside the Pomo Pit). Overall mean coefficients are also reported for each habitat (Within P and Within S)

| **Size class** | **Mean Bray-Curtis dissimilarity** |
| --- | --- |
| < 120 P | 0.75 |
| 150-199 P | 0.13 |
| 200-249 P | 0.66 |
| 250-299 P | 0.83 |
| ≥ 300 S P | 0.30 |
| **Within P** | 0.53 |
| 120-149 S | 0.72 |
| 150-199 S | 0.66 |
| 200-249 S | 0.90 |
| 250-299 S | 0.61 |
| ≥ 300 S | 0.75 |
| **Within S** | 0.73 |

**Table S4**. *Permutest* pairwise comparison among the 10 NC Adriatic groups of *M. merluccius*, identified for each size class/habitat (combination of size class and habitat; P: sample collected within the Pomo Pit; S: individuals collected outside the Pomo Pit). Observed P-value are below diagonal, permuted P-value are above diagonal. Significant values are in bold (significance level of 0.01).

|  | <120 P | 150-199 P | 200-249 P | 250-299 P | ≥ 300 P | 120-149 S | 150-199 S | 200-249 S | 250-299 S | ≥ 300 S |
| --- | --- | --- | --- | --- | --- | --- | --- | --- | --- | --- |
| <120 P |  | 0.1370 | 0.9800 | 0.7300 | 0.1460 | 0.9710 | 0.9550 | 0.9110 | 0.7170 | 0.7320 |
| 150-199 P | 0.1428 |  | **0.004** | **0.001** | 0.3840 | **0.001** | **0.005** | **0.009** | **0.001** | **0.001** |
| 200-249 P | 0.9795 | 0.0037 |  | 0.3900 | 0.0180 | 0.9860 | 0.8480 | 0.7470 | 0.4870 | 0.4110 |
| 250-299 P | 0.7530 | 0.0003 | 0.3900 |  | **0.002** | 0.2620 | 0.5730 | 0.6940 | 0.0880 | 0.8900 |
| ≥ 300 P | 0.1600 | 0.3872 | 0.0138 | 0.0027 |  | **0.006** | 0.0300 | **0.012** | 0.0460 | **0.003** |
| 120-149 S | 0.9816 | 0.0006 | 0.9903 | 0.2599 | 0.0050 |  | 0.8240 | 0.7300 | 0.3780 | 0.2920 |
| 150-199 S | 0.9530 | 0.0089 | 0.8573 | 0.5527 | 0.0269 | 0.8316 |  | 0.9200 | 0.4110 | 0.5790 |
| 200-249 S | 0.9004 | 0.0068 | 0.7665 | 0.6848 | 0.0154 | 0.7336 | 0.9225 |  | 0.3630 | 0.7070 |
| 250-299 S | 0.7307 | 0.0058 | 0.4867 | 0.0830 | 0.0364 | 0.3882 | 0.4224 | 0.3633 |  | 0.0680 |
| ≥ 300 S | 0.7442 | 0.0001 | 0.4023 | 0.9018 | 0.0012 | 0.2933 | 0.5893 | 0.7122 | 0.0825 |  |

**Table S5**. Summary of sampling information of *Merluccius merluccius* individuals collected in 2016 and metabarcoded in this study: specimen ID: individual code; size class/habitat: combination of size class and habitat (P: individuals collected within the Pomo Pit; S: individuals collected outside the Pomo Pit), Shooting latitude and longitude: geographical coordinates; Haul number: MEDITS 2016 haul number; Depth: mean haul depth.

| **Specimen ID** | **Size class/ habitat** | **Shooting latitude** | **Shooting longitude** | **Haul number** | **Depth**  **(m)** |
| --- | --- | --- | --- | --- | --- |
| 10 | < 120 P | 42.751 | 14.854 | 50 | 209 |
| 11 | < 120 P | 42.751 | 14.854 | 50 | 209 |
| 12 | < 120 P | 42.751 | 14.854 | 50 | 209 |
| 13 | < 120 P | 42.751 | 14.854 | 50 | 209 |
| 14 | < 120 P | 42.751 | 14.854 | 50 | 209 |
| 17 | < 120 P | 42.751 | 14.854 | 50 | 209 |
| 27 | < 120 P | 43.384 | 15.471 | 36 | 201 |
| 28 | < 120 P | 43.384 | 15.471 | 36 | 201 |
| 29 | < 120 P | 43.384 | 15.471 | 36 | 201 |
| 3 | 150-199 P | 42.751 | 14.854 | 50 | 209 |
| 4 | 150-199 P | 42.751 | 14.854 | 50 | 209 |
| 5 | 150-199 P | 42.751 | 14.854 | 50 | 209 |
| 6 | 150-199 P | 42.751 | 14.854 | 50 | 209 |
| 8 | 150-199 P | 42.751 | 14.854 | 50 | 209 |
| 9 | 150-199 P | 42.751 | 14.854 | 50 | 209 |
| 15 | 150-199 P | 42.751 | 14.854 | 50 | 209 |
| 16 | 150-199 P | 42.751 | 14.854 | 50 | 209 |
| 25 | 150-199 P | 43.384 | 15.471 | 36 | 201 |
| 26 | 200-249 P | 43.384 | 15.471 | 36 | 201 |
| 48 | 200-249 P | 43.053 | 15.116 | 43 | 260 |
| 81 | 200-249 P | 42.917 | 14.970 | 45 | 194 |
| 83 | 200-249 P | 42.917 | 14.970 | 45 | 194 |
| 84 | 200-249 P | 42.950 | 15.069 | 44 | 212 |
| 86 | 200-249 P | 42.950 | 15.069 | 44 | 212 |
| 97 | 200-249 P | 42.871 | 14.917 | 46 | 206 |
| 98 | 200-249 P | 43.369 | 15.283 | 37 | 235 |
| 103 | 200-249 P | 42.752 | 14.604 | 171 | 164 |
| 22 | 250-299 P | 43.384 | 15.471 | 36 | 201 |
| 43 | 250-299 P | 42.871 | 14.917 | 46 | 206 |
| 49 | 250-299 P | 43.053 | 15.116 | 43 | 260 |
| 85 | 250-299 P | 42.950 | 15.069 | 44 | 212 |
| 101 | 250-299 P | 42.486 | 14.884 | 167 | 151 |
| 102 | 250-299 P | 42.752 | 14.604 | 171 | 164 |
| 107 | 250-299 P | 42.820 | 14.687 | 170 | 233 |
| 1 | ≥ 300 P | 42.751 | 14.854 | 50 | 209 |
| 2 | ≥ 300 P | 42.751 | 14.854 | 50 | 209 |
| 38 | ≥ 300 P | 42.871 | 14.917 | 46 | 206 |
| 39 | ≥ 300 P | 42.871 | 14.917 | 46 | 206 |
| 40 | ≥ 300 P | 42.871 | 14.917 | 46 | 206 |
| 41 | ≥ 300 P | 42.871 | 14.917 | 46 | 206 |
| 42 | ≥ 300 P | 42.871 | 14.917 | 46 | 206 |
| 45 | ≥ 300 P | 42.871 | 14.917 | 46 | 206 |
| 46 | ≥ 300 P | 42.871 | 14.917 | 46 | 206 |
| 63 | 120-149 S | 43.651 | 14.703 | 27 | 79.5 |
| 64 | 120-149 S | 43.651 | 14.703 | 27 | 79.5 |
| 65 | 120-149 S | 43.651 | 14.703 | 27 | 79.5 |
| 72 | 120-149 S | 43.353 | 14.271 | 72 | 78.3 |
| 73 | 120-149 S | 43.353 | 14.271 | 72 | 78.3 |
| 78 | 120-149 S | 44.186 | 14.103 | 83 | 67.1 |
| 79 | 120-149 S | 44.186 | 14.103 | 83 | 67.1 |
| 90 | 120-149 S | 43.970 | 13.585 | 88 | 67.1 |
| 92 | 120-149 S | 43.970 | 13.585 | 88 | 67.1 |
| 57 | 150-199 S | 43.651 | 14.703 | 27 | 79.5 |
| 59 | 150-199 S | 43.651 | 14.703 | 27 | 79.5 |
| 60 | 150-199 S | 43.651 | 14.703 | 27 | 79.5 |
| 61 | 150-199 S | 43.651 | 14.703 | 27 | 79.5 |
| 62 | 150-199 S | 43.651 | 14.703 | 27 | 79.5 |
| 68 | 150-199 S | 43.353 | 14.271 | 72 | 78.3 |
| 69 | 150-199 S | 43.353 | 14.271 | 72 | 78.3 |
| 70 | 150-199 S | 43.353 | 14.271 | 72 | 78.3 |
| 71 | 150-199 S | 43.353 | 14.271 | 72 | 78.3 |
| 53 | 200-249 S | 43.651 | 14.703 | 27 | 79.5 |
| 54 | 200-249 S | 43.651 | 14.703 | 27 | 79.5 |
| 55 | 200-249 S | 43.651 | 14.703 | 27 | 79.5 |
| 56 | 200-249 S | 43.651 | 14.703 | 27 | 79.5 |
| 58 | 200-249 S | 43.651 | 14.703 | 27 | 79.5 |
| 67 | 200-249 S | 43.353 | 14.271 | 72 | 78.3 |
| 76 | 200-249 S | 44.186 | 14.103 | 83 | 67.1 |
| 95 | 200-249 S | 43.970 | 13.585 | 88 | 67.1 |
| 112 | 200-249 S | 43.771 | 14.553 | 26 | 81.5 |
| 75 | 250-299 S | 44.186 | 14.103 | 83 | 67.1 |
| 87 | 250-299 S | 43.970 | 13.585 | 88 | 67.1 |
| 88 | 250-299 S | 43.970 | 13.585 | 88 | 67.1 |
| 91 | 250-299 S | 43.970 | 13.585 | 88 | 67.1 |
| 110 | 250-299 S | 43.771 | 14.553 | 26 | 81.5 |
| 116 | 250-299 S | 43.771 | 14.553 | 26 | 81.5 |
| 121 | 250-299 S | 43.771 | 14.553 | 26 | 81.5 |
| 122 | 250-299 S | 43.771 | 14.553 | 26 | 81.5 |
| 132 | 250-299 S | 43.771 | 14.553 | 26 | 81.5 |
| 52 | ≥ 300 S | 43.651 | 14.703 | 27 | 79.5 |
| 66 | ≥ 300 S | 43.353 | 14.271 | 72 | 78.3 |
| 74 | ≥ 300 S | 44.186 | 14.103 | 83 | 67.1 |
| 130 | ≥ 300 S | 43.771 | 14.553 | 26 | 81.5 |
| 134 | ≥ 300 S | 43.771 | 14.553 | 26 | 81.5 |
| 135 | ≥ 300 S | 44.469 | 13.254 | 21 | 46.3 |
| 136 | ≥ 300 S | 44.469 | 13.254 | 21 | 46.3 |
| 145 | ≥ 300 S | 44.017 | 13.853 | 86 | 70 |
| 148 | ≥ 300 S | 43.833 | 14.435 | 25 | 74.5 |
| 149 | ≥ 300 S | 44.167 | 12.983 | 7 | 44.8 |

**Figure S1**. Species accumulation curves showing species richness (based on all taxa detected) in *M. merluccius* stomach samples collected within the Pomo pit (blue curve) and outside the Pomo pit (black curve). The method “rarefaction” implemented in the *specaccum* function (*vegan* package, R) was used.

**Figure S2**. Left side: bipartite networks depicting diet composition and overlap of the 10 NC Adriatic samples of *M. merluccius*, identified for each size class/habitat (combination of size class and habitat; P: sample collected within the Pomo Pit; S: individuals collected outside the Pomo Pit). Lines connect *M. merluccius* samples (top boxes) to prey sequences (bottom boxes) and are proportional to interaction strength. Right side: heat map showing prey composition of *M. merluccius* diet for each size class (darker colour depict stronger interactions). In red the number of interactions.
